# In vivo confirmation of altered hepatic glucose metabolism in patients with liver fibrosis/cirrhosis by ^18^F-FDG PET/CT

**DOI:** 10.1186/s13550-018-0452-y

**Published:** 2018-11-09

**Authors:** Niklas Verloh, Ingo Einspieler, Kirsten Utpatel, Karin Menhart, Stefan Brunner, Frank Hofheinz, Jörg van den Hoff, Philipp Wiggermann, Matthias Evert, Christian Stroszczynski, Dirk Hellwig, Jirka Grosse

**Affiliations:** 1 Department of Nuclear Medicine, University Hospital Regensburg, Regensburg, Germany; 2 Department of Radiology, University Hospital Regensburg, Regensburg, Germany; 3 Department of Pathology, University Regensburg, Regensburg, Germany; 4 Department of Surgery, University Hospital Regensburg, Regensburg, Germany; 5 Helmholtz-Zentrum Dresden-Rossendorf, PET Center, Institute of Radiopharmaceutical Cancer Research, Dresden, Germany; 6 Department of Radiology and Nuclear Medicine, Hospital Braunschweig, Braunschweig, Germany

**Keywords:** ^18^F-FDG PET/CT, Hepatic metabolism, Liver fibrosis, Liver cirrhosis, FDG kinetics, METAVIR score

## Abstract

**Objective:**

The aim of this study was to assess the value of ^18^F-FDG PET/CT for quantitative assessment of hepatic metabolism in patients with different stages of liver fibrosis/cirrhosis.

**Materials and methods:**

^18^F-FDG PET/CT scans of 37 patients either with or without liver fibrosis/cirrhosis, classified according to the METAVIR score (F0-F4) obtained from histopathological analysis of liver specimen, were analyzed retrospectively and classified as follows: no liver fibrosis (F0, *n* = 6), mild liver fibrosis (F1, *n* = 11), advanced liver fibrosis (F2, *n* = 6), severe liver fibrosis (F3, *n* = 5), and liver cirrhosis (F4, *n* = 11). The liver-to-blood ratio (LBR, scan time corrected for a reference time of 75 min) was compared between patient groups.

**Results:**

Patients with liver fibrosis or cirrhosis (≥ F1; LBR 1.53 ± 0.35) showed a significant higher LBR than patients with normal liver parenchyma (F0, 1.08 ± 0.23; *P* = 0.004). In direct comparison, LBR increased up to the advanced stage of liver fibrosis (F2; 2.00 ± 0.40) and decreased until liver cirrhosis is reached (F4, 1.32 ± 0.14).

**Conclusion:**

Functional changes in liver parenchyma during liver fibrosis/cirrhosis affect hepatic glucose metabolism and significantly differ between stages of liver fibrosis/cirrhosis, classified according to the METAVIR scoring system, as demonstrated by LBR quantification by ^18^F-FDG PET/CT.

## Introduction

The increasing prevalence of chronic liver diseases is a growing problem for the Western Hemisphere, as patients’ morbidity and mortality are directly correlated with the progression of hepatic fibrosis. Today, liver cirrhosis is among the leading causes of mortality in the Western Hemisphere and causes significant health care costs [1–4].

Various imaging modalities, including computed tomography (CT), magnetic resonance imaging (MRI), and ultrasonography (US), are being used to evaluate the liver parenchyma [5–9]. In conventional nuclear medicine, scintigraphy and single-photon emission computed tomography with ^99m^Tc-IDA or ^99m^Tc-GSA can be used to quantify hepatic function and assess liver fibrosis and hepatic functional reserve [10–14]. Apart from that, technically more complex positron emission tomography (PET) is an emerging imaging modality with superior spatial resolution, which is used not only in oncology but increasingly also to visualize infectious, inflammatory, and degenerative diseases [15, 16]. ^18^F-fluoro-2-deoxy-D-glucose (FDG) is the most commonly used radiopharmaceutical for PET examinations. It allows the quantification of glucose metabolism in different tissues.

The quantification of regional glucose metabolism can be achieved by kinetic analysis of FDG uptake of dynamic PET acquisitions or more simply by means of standardized uptake value (SUV) measurements from static PET images [15, 17]. However, the accuracy and reproducibility of the SUV for quantitative analysis of whole-body PET/CT examinations are influenced by both technical and biological factors, leading to a limited test-retest reproducibility of SUV measurements [18]. The tissue-to-blood ratio (TBR) specifies the ratio between an organ and the (aortic) blood pool and is insensitive to cross-calibration errors between PET scanners and dose calibrators [18]. However—like SUV—it does not take into account the biology-driven time dependence of the FDG accumulation, which contributes substantially to incongruent results with respect to comparative studies. For tumors, the tumor-to-blood standard uptake ratio (SUR) was introduced to overcome both technical and biological factors, potentially leading to a more accurate quantification of FDG uptake and thus of glucose metabolism [19]. For tissue analysis, the correction of SUV for the scan time interval results in improved comparability of PET examinations acquired at different times after FDG injection [20]. The correction used for SUR calculations is based mainly on the observations that, on the one hand, the FDG concentration in arterial blood exhibits a hyperbolic decline pattern, and on the other hand, the tumor-to-blood ratio over time becomes almost linear after tracer equilibration in the blood. Unlike with malignant tumors, the uptake of FDG into the liver does not follow an irreversible kinetics. It exhibits a low and homogeneous uptake of ^18^F-FDG into the liver parenchyma [21–24].

To what extent FDG uptake is affected by diffuse liver diseases is not clear yet. Hernandez-Martinez et al. reported that the FDG uptake was reduced in cirrhotic livers (defined by anatomical imaging criteria) compared to the control group [25]. Some studies, evaluating the ^18^F-FDG uptake in liver steatosis, reported an increasing SUV compared to normal liver parenchyma, whereas others observed no such difference [26–28]. In addition, there is evidence that a higher hepatic glucose metabolism with higher ^18^F-FDG uptake into the liver parenchyma is associated with increased expression of GLUT-1 and hexokinase II [29, 30].

For assessing liver inflammation in non-alcoholic steatohepatitis (NASH), a recent study showed that the dynamic ^18^F-FDG-PET with kinetic modeling has the potential to assess liver inflammation in patients with NASH, while hepatic glucose metabolism assessed by means of SUV-analyzes gave no promising results [31].

The precise assessment of hepatic ^18^F-FDG accumulation and distribution could be essential, considering the unmet need for noninvasive staging of chronic liver diseases. Given the potential role of molecular imaging in the assessment of hepatic disorders, the aim of this study was to evaluate hepatic glucose metabolism in patients with different stages of liver fibrosis/cirrhosis by means of ^18^F-FDG PET/CT.

## Materials and methods

### Patients

We retrospectively analyzed 37 consecutive ^18^F-FDG PET/CT scans and corresponding histopathological liver samples obtained between 11/2008 and 09/2017. ^18^F-FDG PET/CT was performed for staging and follow-up of malignant diseases, whereas biopsy of the liver was done due to known liver fibrosis/cirrhosis or suspicious liver lesion. Patients were included if the timespan between ^18^F-FDG PET/CT and histopathological examination did not exceed 6 months. Patients treated with chemotherapy or radiation therapy during the 6 months prior to FDG-PET/CT imaging were excluded. Patient characteristics are shown in Table 1.

**Table 1:** Patients characteristics. Continuous measures are reported as mean with the corresponding standard deviation.

Male [*n* (%)]	22 (60)
Age [years]	61 ± 13
Height [m]	1.71 ± 0.07
Weight [kg]	81 ± 18
BMI [kg/m^2^]	27.5 ± 6.2
Blood glucose level [mg/dl]	112 ± 25

*BMI* body mass index

Approval from the local ethics committee of the University Hospital Regensburg was obtained, and this retrospective study was performed in accordance with all relevant guidelines and regulations.

### Imaging

^18^F-FDG PET/CT imaging was performed using a Biograph 16 PET/CT scanner (CTI-Siemens, Erlangen, Germany) consisting of a 16-slice multidetector CT (0.5 s per revolution) and a PET detector with an axial and transaxial field-of-view of 162 mm and 585 mm, respectively.

After a fasting period of at least 6 h, 3 MBq ^18^F-FDG per kilogram body weight were injected intravenously (321 ± 48 MBq).The patients’ blood glucose level was strictly controlled to be below 150 mg/dL (8.32 mmol/L). To increase renal tracer elimination, they received an injection of 20 mg furosemide as well as oral or intravenous hydration shortly after ^18^F-FDG injection. In order to minimize muscular ^18^F-FDG uptake, patients were advised to stay in a quiet lying position. Warming blankets were used to avoid freezing of the patients and to keep potential tracer accumulation in brown fat tissue to a minimum. Patients were instructed to void the bladder prior to scanning and to remove all metal parts.

After a waiting period of about 60 min post-injection (minimum 50 min, maximum 90 min), the PET/CT acquisition was performed with elevated arms to acquire images of the trunk (pelvis to skull base). Depending on the patient size and clinical indication, six to eight overlapping bed positions with 3 min of PET acquisition time each were used. The same area was covered by a low-dose CT scan (tube current 50 mAs, tube voltage 120 keV) if no contrast agents were used. In 17 patients, intravenous contrast agent (130 ml of Accupaque™ 300, GE Healthcare) was applied with consecutive full-dose CT acquisition (120 keV, 100 mAs).

PET images (slice thickness 5 mm) were corrected for random coincidences, decay, scatter, and attenuation and reconstructed iteratively using the ordered subsets expectation maximization algorithm (OSEM) with four iterations and eight subsets. PET images were scaled to allow SUV measurements. PET and CT images were checked for breathing artifacts.

### Image analysis

Images were interpreted by a nuclear medicine physician and a radiologist. To determine the SUV_mean_ in the liver parenchyma, a 3D volume of interest (VOI) was placed manually in the right liver lobe, excluding visible vessels and liver lesions. VOI size ranged from 13.6 to 17.2 cm^3^. To control for the potential interindividual differences in overall glucose metabolism, VOIs with a diameter of 1 cm were placed in the gluteal muscle to determine the SUV_mean_. The aortic blood pool SUV was determined by delineating the aorta in the attenuation CT. The resulting intraluminal ROI was then transferred to the coregistered PET image and the mean value was set as the blood pool value. Tissue-to-blood ratios (TBR, Eq. 1) and the time corrected liver-to-blood ratio (LBR, Eq. 2) were calculated using the following formulas:

1 $$ \mathrm{Tissue}-\mathrm{to}-\mathrm{blood}\ \mathrm{ratio}\ (T)\ \left(\mathrm{TBR}\right)=\frac{\mathrm{SUV}(T)\ {\mathrm{mean}}_{\mathrm{Target}}}{\mathrm{SUV}(T)\ {\mathrm{mean}}_{\mathrm{Blood}\ \mathrm{pool}}} $$

2 $$ \mathrm{Liver}-\mathrm{to}-\mathrm{blood}\ \mathrm{ratio}\ \left(T,{T}_0\right)\ \left(\mathrm{LBR}\right)={\left(\frac{T_0}{T}\right)}^b\times \frac{\mathrm{SUV}(T)\ {\mathrm{mean}}_{\mathrm{Target}}}{\mathrm{SUV}(T)\ {\mathrm{mean}}_{\mathrm{Blood}\ \mathrm{pool}}}={\left(\frac{T_0}{T}\right)}^b\times \mathrm{TBR}(T), $$

with *T*_0_: reference time (75 min), *T*: acquisition time, and *b*: correction factor (set to 0.313 according to [20]).

### Histopathological examination

For the histopathological examination, liver biopsies or partial resections were used. The length of each biopsy specimen was measured, and the number of portal tracts was assessed. The liver samples were included in the evaluations only when the tissue length exceeded 15 mm and more than ten portal tracts were visible. Only non-tumorous liver tissue was included in this study. All samples were fixed in formalin and embedded in paraffin. Four-micrometer sections were cut vertically and mounted on glass slides. The sections were deparaffinized with xylene and ethanol and stained with hematoxylin-eosin (HE) and Elastica van Gieson (EVG) according to standard protocols. EVG staining was used to evaluate the liver fibrosis with collagen stained red and hepatocytes stained yellow.

Two pathologists (M.E. and K.U.), who specialize in liver histopathology, assessed/evaluated/graded the samples’ degrees of fibrosis/cirrhosis using the METAVIR scoring system [32, 33]. Both readers were blinded to the imaging results and the patient data. The scoring was performed independently. In cases of disagreement, additional microscopic analyses were performed and a common final judgment was made in consensus. The patients were subdivided into the following five categories: F0 (*n* = 6) no fibrosis, F1 (*n* = 11) mild fibrosis, F2 (*n* = 6) advanced fibrosis, F3 (*n* = 6) severe fibrosis, and F4 (*n* = 11) cirrhosis.

### Statistical analysis

All statistical analyses were performed with IBM SPSS Statistics (version 24, Chicago, IL, USA). The data are presented as mean ± standard deviation (SD). Non-parametric Mann-Whitney *U* test for independent variables were used to compare groups. All tests were two-sided and a significance level of *p* < 0.05 was considered significant.

## Results

**Fig. 1:**
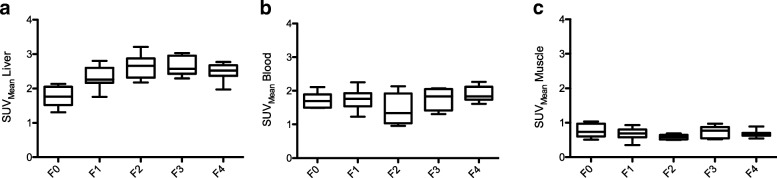
The standardized uptake values (SUV) of the liver parenchyma (**a**), the aortic blood pool (**b**), and the skeletal muscle (**c**) in patients with normal liver parenchyma and patients with liver fibrosis/cirrhosis are shown. The Mann-Whitney *U* test was used to compare the following groups: no (F0), mild (F1), advanced (F2), severe liver fibrosis (F3), and liver cirrhosis (F4)

Patients with liver fibrosis/cirrhosis (≥ F1; SUV_Liver_ 2.48 ± 0.32) showed a significantly higher SUV of the liver parenchyma (SUV 2.48 + − 0.32) than patients with normal liver parenchyma (F0; SUV_Liver_ 1.76 ± 0.31; *p* < 0.001), while the SUV of the skeletal muscle did not differ between the groups (Table 2). Figure 1 shows the SUVs of the liver parenchyma, aortic blood pool, and skeletal muscle in patients with liver fibrosis/cirrhosis and patients with normal liver parenchyma.

**Table 2:** This table shows the mean standardized uptake values (SUV) of the liver parenchyma, aortic blood pool, and skeletal muscle with their corresponding tissue-to-blood ratios (TBR), as well as the time corrected liver-to-blood-ratio (LBR) for patients with normal liver parenchyma and patients with different stages of liver fibrosis according to the METAVIR classification

	Mean SUV blood	Mean SUV liver	Mean SUV muscle	TBR_Liver_	TBR_Muscle_	LBR
No liver fibrosis (F0, *n* = 6)	1.72 ± 0.23	1.76 ± 0.31	0.77 ± 0.20	1.04 ± 0.23	0.44 ± 0.08	1.08 ± 0.23
Mild liver fibrosis (F1, *n* = 11)	1.75 ± 0.31	2.31 ± 0.31	0.74 ± 0.25	1.35 ± 0.25	0.42 ± 0.16	1.44 ± 0.27
Advanced liver fibrosis (F2, *n* = 6)	1.45 ± 0.46	2.64 ± 0.36	0.58 ± 0.07	1.93 ± 0.40	0.43 ± 0.09	2.00 ± 0.40
Severe liver fibrosis (F3, *n* = 5)	1.75 ± 0.33	2.62 ± 0.29	0.72 ± 0.18	1.55 ± 0.23	0.41 ± 0.06	1.53 ± 0.20
Liver cirrhosis (F4, *n* = 11)	1.90 ± 0.22	2.49 ± 0.24	0.68 ± 0.10	1.32 ± 0.15	0.36 ± 0.05	1.32 ± 0.14

**Fig. 2:**
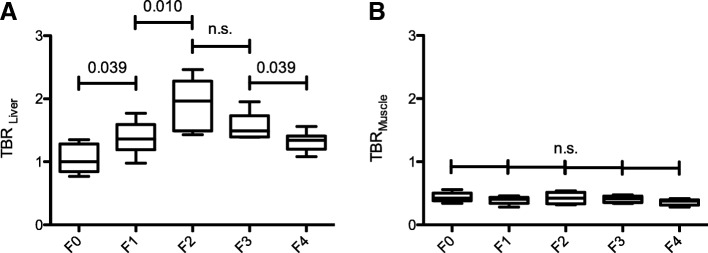
Shows the tissue-to-blood ratios (TBRLiver, (**a**); TBRMuscle, (**b**)) in patients with normal liver parenchyma and patients with liver fibrosis/cirrhosis. The Mann-Whitney *U* tests was used to compare the groups: no (F0), mild (F1), advanced (F2), severe liver fibrosis (F3), and liver cirrhosis (F4)

In the non-time-corrected TBR analysis, a comparison between patients with and without liver fibrosis/cirrhosis as stratified according to the METAVIR scoring system revealed that TBR_Liver_ increased up to advanced liver fibrosis (F2; TBR_Liver_ 1.93 ± 0.40) and then decreased until liver cirrhosis is reached (Table 2). Compared to the altered glucose metabolism of the liver parenchyma, no significant difference of TBR for the skeletal muscle was found. Figure 2 shows the boxplots of the ratio between the mean SUV in the liver tissue (TBR_Liver_) and in the skeletal muscle (TBR_Muscle_), corrected with the aortic blood pool, in patients with normal liver parenchyma and patients with liver fibrosis/cirrhosis and the corresponding significance values for the analyzed patients.

Patients with liver fibrosis/cirrhosis (≥ F1) showed a significantly higher LBR (1.53 ± 0.35) than patients with normal liver parenchyma (F0; 1.08 ± 0.23; *p* = 0.004). In pairwise comparison, significant differences in LBR were observed between patients without fibrosis (F0) and those with mild liver fibrosis (F1; LBR 1.44 ± 0.27; *p* = 0.035) and between patients with initial and those with advanced liver fibrosis (F2; LBR 2.00 ± 0.40; *p* = 0.015). A significant difference was observed between the patients with advanced and severe liver fibrosis (F3; LBR 1.53 ± 0.20; *p* = 0.028). No significant difference was observed between patients with severe liver fibrosis and liver cirrhosis (F4; LBR 1.32 ± 0.14; *p* = 0.053). However, a *p* value of 0.053 indicates a trend towards significance.

**Fig. 3:**
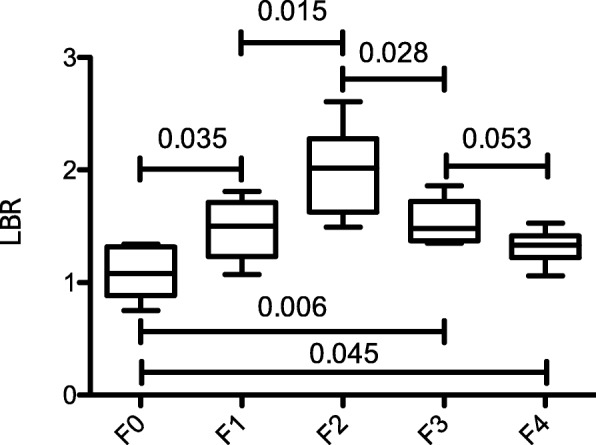
LBR in patients with normal liver parenchyma and patients with liver fibrosis/cirrhosis. Mann-Whitney *U* tests were used to compare the groups: no (F0), mild (F1), advanced (F2), severe liver fibrosis (F3), and liver cirrhosis (F4)

Figure 3 and Table 3 show the boxplots and the corresponding significance values for the analyzed patients.

**Table 3:** Differentiation between the stages of liver fibrosis

	F0	F1	F2	F3	F4
F0		0.035	0.002	0.006	0.045
F1	0.035		0.015	0.571	0.362
F2	0.002	0.015		0,028	0.001
F3	0.006	0.571	0.028		0.053
F4	0.045	0.362	0.001	0.053	

Comparison of the different stages of liver fibrosis, stratified by the METAVIR scoring system, with the corresponding *p* values: no (F0), mild (F1), advanced (F2), severe liver fibrosis (F3), and liver cirrhosis (F4)

## Discussion

Liver cirrhosis is characterized by nodular regeneration of liver tissue with the destruction of the lobular and vascular architecture [34–36]. The progression of liver fibrosis and the development of liver cirrhosis are currently viewed as a dynamic process [37]. In the development of inflammation and fibrosis, different factors such as oxidative stress, mitochondrial changes, and hormonal disorders are taken into account as factors [38–40]. With increased tissue remodeling during wound healing processes, one can assume higher glucose metabolism in the liver parenchyma.

**Fig. 4:**
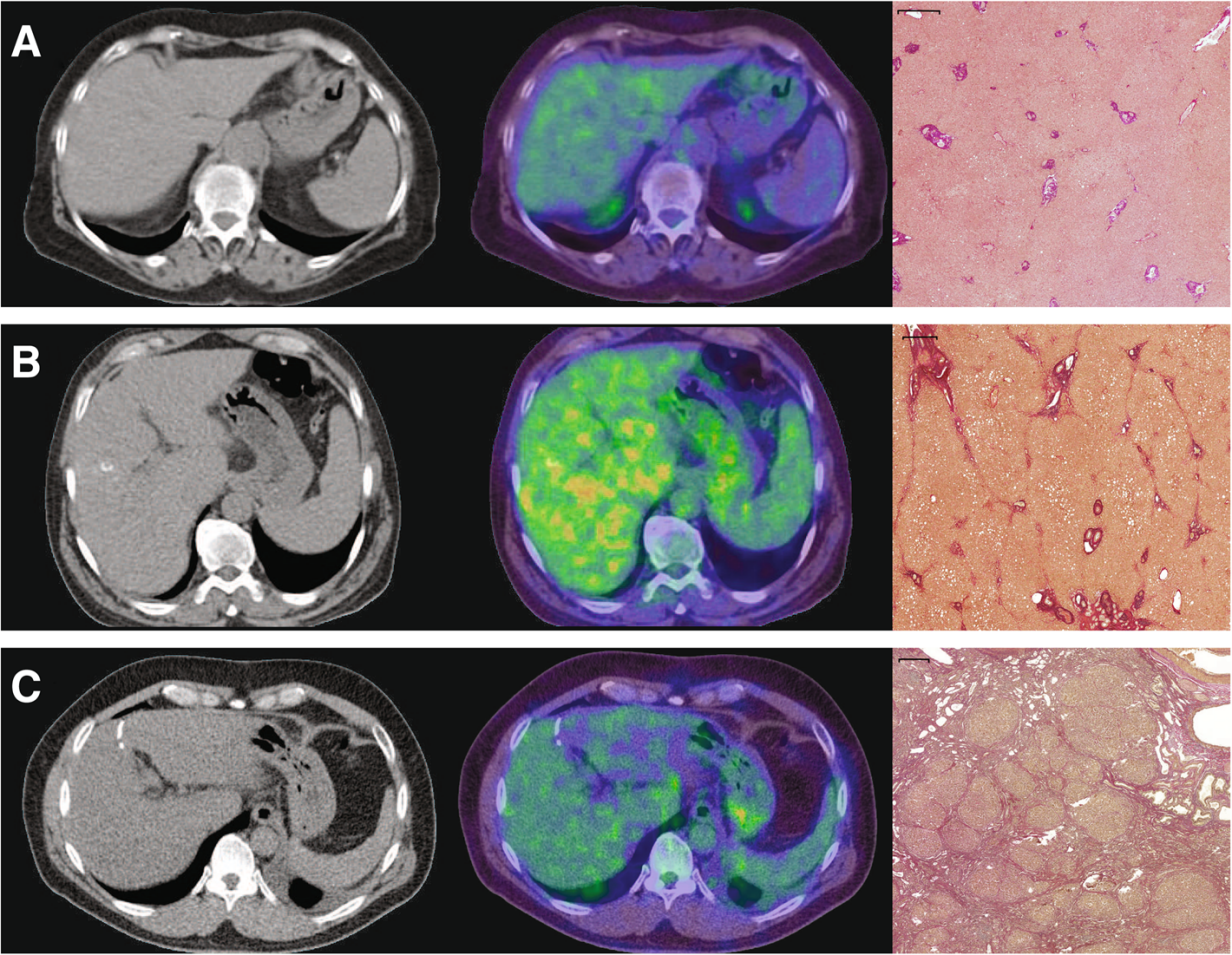
CT- (left column) and fused PET/CT-images (middle column) of patients with normal liver parenchyma (**a**), advanced liver fibrosis (**b**), and with liver cirrhosis (**c**) with the corresponding histopathology images demonstrating EVG staining (right column). CT images are displayed with the same window (400) and center (40) level. The fused PET/CT-images are shown with an upper SUV threshold of 7.0 using the spectrum color lookup table (SyngoVia) for PET. The scale on histological slices represents 500 μm

We observed that the TBR and the LBR of the liver increase with advanced liver fibrosis (F1 to F2). In patients with severe liver fibrosis (F3) or liver cirrhosis (F4), the LBR then decreases compared to patients with advanced liver fibrosis, indicating a higher activity level of tissue remodeling in patients with advanced liver fibrosis (Fig. 4). These findings are in accord with the morphological changes in the expression of GLUT in the liver parenchyma because hepatocytes are capable of gluconeogenesis and their need for glucose uptake is modest [41]. In normal liver parenchyma, all GLUTs are expressed, except for GLUT-7 [42], and the liver parenchyma shows overexpression of GLUT-2, − 8, − 9, and − 10 [43]. GLUT-1 and GLUT-2 allow for efficient uptake of glucose at low plasma glucose concentrations [44]. Expression of these transporters in liver cells is restricted to hepatocytes proximal to the hepatic venule [45]. In damaged liver parenchyma, the majority of GLUTs are upregulated compared to normal liver parenchyma [43] and the expression of GLUT-1 in hepatocytes is increased, while GLUT-2 is decreased [46]. While GLUT-2 ensures, independently of insulin, with its low affinity and high transport capacity, that intracellular and extracellular glucose concentrations are in equilibrium [47], GLUT-1 has a higher affinity for glucose and is nearly saturated under physiological conditions [48]. In addition, the amine oxidase activity of VAP-1 is upregulated in chronic liver diseases [49]. VAP-1 plays a significant role in glucose uptake into hepatocytes as it stimulates glucose uptake via translocation of transporters to the cell membrane [43, 50]. In conclusion, the high affinity of GLUT-1 for glucose in combination with its increased expression in damaged liver parenchyma might explain the increased ^18^F-FDG uptake we observed in patients with liver fibrosis suggesting a higher glucose uptake for liver cells.

Non-parenchymal liver cells, which cannot carry out gluconeogenesis, rely on glucose uptake rather than on endogenous formation. GLUT-1 is the dominant transporter protein in both endothelial cells and Kupffer cells, and its expression levels increase during inflammation induced by lipopolysaccharide [51].

In the process of tissue remodeling in liver parenchyma, inflammation plays an important role [52–54]. As a reaction to damaged hepatocytes, apoptotic bodies will be recruited to interact with quiescent hepatic stellate cells and Kupffer cells to activate and promote inflammatory and fibrogenic responses [55, 56]. As a consequence, the enhanced inflammatory and immune-mediated responses will promote hepatocyte necrosis and apoptosis, which nurtures further fibrogenic processes [57].

One may argue that Kupffer cells are more dominant in patients with higher levels of active tissue remodeling, as it is observed for patients with advanced liver fibrosis (F2). This might explain the increased glucose metabolism in patients with advanced liver fibrosis in addition to elevated ^18^F-FDG uptake in damaged hepatocytes as described above.

In our study, patients with severe liver fibrosis (F3) or liver cirrhosis (F4) showed a decreased glucose metabolism compared to patients with advanced liver fibrosis (F2), while maintaining a higher glucose metabolism than patients with normal liver parenchyma (F0) (Fig. 3). These results are in contrast to Hernandez-Martinez et al., who reported a reduced FDG uptake in cirrhotic livers in comparison to the control group [25]. However, Hernandez-Martinez et al. [25] used a combination of clinical, histopathological, and imaging data for the classification of liver disease, whereas we used a validated histopathological scoring system, the METAVIR scoring system. Furthermore, they used the error-prone SUV measurements, in contrast to our scan time corrected LBR quantification.

There are some potential biological explanations for our observations. The decreased glucose metabolism in patients with liver cirrhosis compared to patients with liver fibrosis corresponds to reports that cirrhotic liver tissue has a depleted glycogen storage [58]. Furthermore, collagen accumulation in liver fibrosis may be associated with reduced uptake of glucose into the liver tissue [59], as observed here. GLUT-4 transporters were detected in sinusoidal endothelial cells as well as in stellate cells where they can mediate glucose uptake by semi-carbazide-sensitive amine oxidase [60] and may contribute to the fibrogenesis in patients with chronic liver diseases. This might contribute to the increased FDG uptake of active fibrosis (F2). In contrast, the expression level of GLUT-4 was found to be decreased in liver cirrhosis [61]; these findings are in line with our data, showing a significantly decreased FDG uptake for patients with METAVIR score F4 in comparison to F2.

These changes in glucose metabolism were solely related to the liver as we can confirm by our observations. In contrast, the skeletal muscle showed no significant difference in glucose metabolism for patients with and without liver fibrosis/cirrhosis.

In direct comparison, quantitative measurements by TBR_Liver_ showed similar findings as LBR. However, the variance in the analyzed subgroups was lower for LBR, resulting in a significant difference in the pairwise comparison of F0, F1, F2, and F3, as well as a trend towards significance between F3 and F4.

There is growing evidence that time-dependent measurements of tumor tissue lead to more exact estimations of metabolic rates of glucose than conventional SUV quantification [62, 63]. While TBR displays the ratio at the time point of acquisition, LBR is computed as the ratio of SUV_Liver_ and SUV_Blood_ with a scan time correction to a reference time (75 min), which accounts for the time-dependent blood SUV, but presently does not try to account for a possible time dependence of the liver SUV in the considered time window.

In most cells, the metabolism of ^18^F-FDG stops after phosphorylation to ^18^F-FDG-6-phosphate, i.e., irreversible kinetics. Therefore, the modeling of the ^18^F-FDG kinetics usually contains three rate constants k1, k2, and k3, whereas hepatocytes contain glucose-6-phosphatase capable of dephosphorylating ^18^FDG-6-phosphate resulting in reversible kinetics with non-negligible k4 [23]. Kinetics of ^18^F-FDG in liver parenchyma is mainly determined by k1 and k2, with a minor impact of k3 and k4 in short-term studies lasting about 60 to 90 min post-FDG injection [31, 64]. In line with the glucose kinetics, SUV_Liver_ in normal liver parenchyma varies little between 60 and 120 min post-injection [65, 66], while the blood activity slowly decreases with time in a hyperbolic manner, i.e., proportional to 1/T^0.313) [20].

However, one must note that the kinetics of fibrotic/cirrhotic tissue is not yet fully understood; a dynamic acquisition at different time points could reveal the actual time dependence of FDG uptake in fibrotic liver parenchyma. Due to the high methodological effort, such an analysis is reserved for a prospective study. In our retrospective study using clinical routine data, PET measurements were only available from static PET scans. With the scan time correction used here, significant disadvantages of conventional SUV quantification, such as cross-calibration errors and time dependence of the FDG distribution, are partially overcome.

Our study has some further limitations. First, the number of patients included in this study was limited, especially for the different small subgroups of liver fibrosis and cirrhosis. We used distribution-free statistical tests to account for this drawback. Second, the accepted time span of 6 months between ^18^F-FDG PET/CT and liver biopsy may be considered rather long. However, as liver fibrosis advances rather slowly, the time span should be tolerable. In prospective studies, a shorter time span would be appreciated. Another limitation might be the sampling error of the liver biopsy: histopathological samples were received from distinct liver areas which might not represent the overall hepatic status.

## Conclusion

In conclusion, we were able to show different activity levels of hepatic glucose metabolism during the process of liver fibrosis and cirrhosis as demonstrated by ^18^F-FDG uptake with PET/CT imaging. These findings highlight the potential of noninvasive molecular imaging for estimating progression and activity of liver fibrosis.

## Abbreviations

CT: Computed tomography
EVG: Elastica van Gieson
FDG: F-fluoro-deoxy-D-glucose
HE: Hematoxylin-eosin
LBR: Liver-to-blood ratio
MRI: Magnetic resonance imaging
NASH: Non-alcoholic steatohepatitis
OSEM: Ordered subsets expectation maximization
PET: Positron emission tomography
SD: Standard deviation
SUR: Standard uptake ratio
SUV: Standardized uptake value
TBR: Tissue-to-blood ratio
US: Ultrasonography
VOI: Volume of interest
